# Bronchiectasis as a complex syndrome: the importance of local registries to overcome heterogeneities

**DOI:** 10.36416/1806-3756/e20250265

**Published:** 2026-03-05

**Authors:** Rodrigo Abensur Athanazio, Liana Sousa Coelho, Samia Zahi Rached, Miguel Ángel Martínez-García

**Affiliations:** 1. Divisao de Pneumologia, Instituto do Coracao, Hospital das Clinicas - HCFMUSP - Faculdade de Medicina, Universidade de Sao Paulo, Sao Paulo (SP) Brasil.; 2. Disciplina de Pneumologia, Departamento de Clínica Médica, Faculdade de Medicina de Botucatu, Universidade Estadual Paulista - UNESP - Botucatu (SP) Brasil.; 3. Servicio de Neumología, Hospital Universitari i Politècnic La Fe, Valencia, España.

Clinical research progresses through the systematic analysis of data collected from patients, including both clinical characteristics and results from complementary diagnostic tests. Randomized controlled trials and meta-analyses consistently generate the highest levels of scientific evidence. Nevertheless, researchers continue to make significant contributions through well-designed prospective and retrospective studies involving large patient cohorts. Among these, national and international disease registries particularly stand out. The aim of this editorial is to highlight the importance of patient registry data in guiding the management of individuals with bronchiectasis, while emphasizing the relevance of establishing a local registry in Brazil.

Patient registries typically consist of large databases that must meet several essential criteria. First, they ideally focus on diseases with low prevalence, allowing researchers to gather sufficient cases to draw meaningful conclusions. Second, the number of variables collected per patient must strike a balance: too many can overburden researchers and hinder data collection, while too few may lead to the omission of critical information needed for future study design. Moreover, these registries must accurately represent the geographic population and preferably follow a longitudinal rather than a cross-sectional approach. Researchers must collect data systematically, completely, and reliably. By design, registries support historical (retrospective) cohort analyses. Nonetheless, they remain crucial for generating hypotheses for future studies, whether prospective observational studies or clinical trials, and for conducting epidemiological investigations.^1^

Bronchiectasis clearly illustrates the value of robust registry data. As the third most common chronic inflammatory airway disease after COPD and asthma, bronchiectasis remains significantly less prevalent, with estimates ranging from 50 to 1,000 cases per 100,000 population, such numbers to be likely underestimated.^2^

Currently, 16 bronchiectasis registries operate worldwide, including the European registry (EMBARC), the Chinese registry (which includes 16,000 patients), and several others that have each surpassed 2,000 patients-such as the Spanish, American, Indian, German, Italian, Australian, Japanese, Turkish, and British registries.^3^

Spain pioneered the establishment of a global bronchiectasis registry including the Spanish historical registry, which ran from 2002 to 2011, and enrolled 2,123 patients from 36 centers. Spain remains unique in maintaining two separate registries. In addition to the historical registry, the country also manages the current online bronchiectasis registry (RIBRON), which has enrolled 2,631 patients from 42 centers and follows them for a median duration of 7 years. Since its inception in 2015, RIBRON has continued to accept both cross-sectional and longitudinal data.^4^ Over the past five years, researchers have published more than 20 international papers using data from these Spanish registries. Their combined analyses offer valuable insights into the evolution of bronchiectasis in Spain over the last two decades.^5^

The European Bronchiectasis Registry (EMBARC) is the only multinational database on bronchiectasis, encompassing data from 28 countries and including over 22,000 patients as of May of 2025. A recent analysis of 16,963 patients highlighted the substantial heterogeneity of the disease across Europe. Among chronic infections, *Pseudomonas aeruginosa* and *Haemophilus influenzae* consistently emerge as the most prevalent pathogens, although their distribution exhibits considerable variation according to the European region assessed. Eastern European countries reported higher rates of exacerbation requiring hospitalization. Treatment approaches differ markedly between countries, underscoring the lack of a universally accepted standard of care,^6^ despite the prior publication of a European consensus.^7^

The growing interest in bronchiectasis has facilitated new discoveries, including identifying inflammatory endotypes driven by either neutrophils or Th2 cells. Advances in understanding the disease pathophysiology have enabled the development of novel therapeutic agents, the cathepsin C inhibitors, such as brensocatib and BI 1291583, with recently published results from phase 3^8^ and phase 2^9^ clinical trials, respectively. These insights also paved the way for exploring biologic therapies targeting type 2 inflammation in bronchiectasis.^10^ An expanding body of evidence now supports the use of inhaled antibiotics, macrolides, and mucoactive agents in managing the disease.^6^

Although the prevalence of bronchiectasis in Brazil remains unknown, its burden is likely substantial. Considering the unequal healthcare access throughout Brazil’s vast territory and the high prevalence of infections, such as tuberculosis and childhood respiratory infections, it is essential to have a Brazilian bronchiectasis registry to understand our patients and their needs. Furthermore, the country has few specialized centers for bronchiectasis, with little information about the characteristics and management of patients.^11^

Given the complexity and diversity of bronchiectasis, it is crucial to understand its characteristics in various contexts to ensure proper patient care, effective treatment strategies, and the development of suitable public policies. Research conducted in Brazil has revealed distinct characteristics of patients with bronchiectasis. A study evaluating Brazilian patients to validate the FACED score in Latin America highlighted these unique traits. Brazilian patients are significantly younger than are those in European and North American publications and exhibit more severe lung disease, with a higher prevalence of *P. aeruginosa* chronic infection.^12^ Although some national data on bronchiectasis is already available, the implementation of a comprehensive national registry still faces significant challenges. These include securing sustainable funding, addressing regional inequalities in healthcare delivery, and overcoming limitations in digital infrastructure that may hinder data collection and integration across the country.

Despite the difficulties in creating a national disease registry, adequate examples have been developed and implemented in Brazil. The Brazilian Cystic Fibrosis (CF) registry is an important initiative, with more than 50 specialized centers and over 6,000 patients. It was essential to incorporate medications into the Brazilian universal health care system and guide the Ministry of Health’s treatment protocol recommendations. Furthermore, data from the Brazilian CF registry allowed for essential publications, such as adequate genotypic characterization of the disease in the country and identifying risk factors for a worse prognosis in our setting.^1^

Given this scenario, a national bronchiectasis registry is long overdue, and it is time to take action. Such a registry would benefit patients by improving the organization and structuring of specialized treatment centers and increasing awareness of the disease among pulmonologists and other healthcare professionals. The heterogeneity of bronchiectasis highlights the need for further research into the underlying causes and mechanisms that lead to a highly diverse microbiota across countries, more effective evidence-based treatments, and improvements in care, especially in regions where bronchiectasis is still considered a neglected disease. Given this pressing need, we must come together to create and implement a national bronchiectasis registry in Brazil. To move forward, we call upon the existing Brazilian centers of excellence in bronchiectasis to lead the formation of a national consortium, with the active engagement of local and national respiratory societies, universities, and governmental agencies, alongside support from both public and private funding sources, to ensure the consolidation and sustainability of a national bronchiectasis registry.

## References

[1] Rached S, Patino CM, Ferreira J (2024). Using data from patient registries to answer important research questions. J Bras Pneumol.

[2] Nigro M, Laska IF, Traversi L, Simonetta E, Polverino E (2024). Epidemiology of bronchiectasis. Eur Respir Rev.

[3] Oscullo G, Bekki A, de la Rosa D, Martinez-García MA (2025). Registries for bronchiectasis in the world an opportunity for international collaboration. Int J Tuberc Lung Dis.

[4] de la Rosa-Carrillo D, Máiz-Carro L, Martínez-García MÁ (2023). What Have We Learned About Bronchiectasis From RIBRON (Spanish Bronchiectasis Registry). Arch Bronconeumol.

[5] Martínez-García MÁ, Oscullo G, Gómez-Olivas JD, Olveira C, Girón R, García-Clemente M (2023). Bronchiectasis Changes in the Characterization of Patients During 20 Years of Follow-up. Data from the Spanish Bronchiectasis Registries. Arch Bronconeumol.

[6] Chalmers JD, Polverino E, Crichton ML, Ringshausen FC, De Soyza A, Vendrell M (2023). Bronchiectasis in Europe data on disease characteristics from the European Bronchiectasis registry (EMBARC). Lancet Respir Med.

[7] Polverino E, Goeminne PC, McDonnell MJ, Aliberti S, Marshall SE, Loebinger MR (2017). European Respiratory Society guidelines for the management of adult bronchiectasis. Eur Respir J.

[8] Chalmers JD, Burgel PR, Daley CL, De Soyza A, Haworth CS, Mauger D (2025). Phase 3 Trial of the DPP-1 Inhibitor Brensocatib in Bronchiectasis. N Engl J Med.

[9] Chalmers JD, Shteinberg M, Mall MA, O'Donnell AE, Watz H, Gupta A (2024). Cathepsin C (dipeptidyl peptidase 1) inhibition in adults with bronchiectasis AIRLEAF(r), a Phase II randomised, double-blind, placebo-controlled, dose-finding study. Eur Respir J.

[10] Bendien SA, Kroes JA, van Hal LHG, Braunstahl GJ, Broeders MEAC, Oud KTM (2023). Real-World Effectiveness of IL-5/5Ra Targeted Biologics in Severe Eosinophilic Asthma With Comorbid Bronchiectasis. J Allergy Clin Immunol Pract.

[11] Pereira MC, Athanazio RA, Dalcin PTR, Figueiredo MRF, Gomes M, Freitas CG (2019). Brazilian consensus on non-cystic fibrosis bronchiectasis. J Bras Pneumol.

[12] Athanazio R, Pereira MC, Gramblicka G, Cavalcanti-Lundgren F, de Figueiredo MF, Arancibia F (2017). Latin America validation of FACED score in patients with bronchiectasis an analysis of six cohorts. BMC Pulm Med.

